# Experience of an Orthopaedic Surgery Department Early During the COVID-19 Outbreak in Japan Including Real-Time Polymerase Chain Reaction Assay Results for SARS-CoV-2

**DOI:** 10.7759/cureus.11140

**Published:** 2020-10-24

**Authors:** Gen Inoue, Kentaro Uchida, Kensuke Fukushima, Katsufumi Uchiyama, Toshiyuki Nakazawa, Jun Aikawa, Terumasa Matsuura, Masayuki Miyagi, Naonobu Takahira, Masashi Takaso

**Affiliations:** 1 Orthopaedic Surgery, Kitasato University, Sagamihara, JPN; 2 Rehabilitation, Kitasato University, Sagamihara, JPN

**Keywords:** covid-19, rt-pcr, orthopaedic surgery

## Abstract

Introduction

The coronavirus disease 2019 (COVID-19) epidemic beginning December 2019 in China has now become a worldwide pandemic. With the need to develop an approach to manage orthopaedic surgeries, we aimed to evaluate the most current data on all the surgical cases in our department including the results of the reverse-transcriptase polymerase chain reaction (RT-PCR) assay for infection with severe acute respiratory syndrome coronavirus 2 (SARS-CoV-2).

Methods

The monthly number of surgical cases from 2016 were reviewed, and compared the numbers of surgical cases both in elective and emergency surgery during the pandemic with the pre-pandemic period. The results of RT-PCR for SARS-CoV-2 in 94 orthopaedic surgery cases from May 13 to June 30, 2020, and clinical signs/symptoms, and laboratory data of 48 consecutive cases within a month from May 13 were also evaluated.

Results

The mean monthly number of surgeries from January to May 2020 was significantly lower than the mean number in 2019 (73.8 vs 121.9, respectively, p=0.01). The proportion of emergency surgeries in all surgeries performed in May 2020 was 35.5%, which is significantly more than the mean rate of 20.4% in 2019 (p=0.04). Hip arthroplasties and spine surgeries showed the greatest reduction, at greater than 80% and 65%, respectively. Although none of the 94 patients were positive for SARS-CoV-2, 66.7% showed signs/symptoms typical of COVID-19. The most frequent signs/symptoms were production of nasal mucus (25.5%), followed by dry cough (19.1%); and fatigue, headache, and dizziness (17.0% each). The incidence of abnormal values, which are commonly noted in COVID-19 patients, were eosinopaenia 37.5%; lymphopaenia 18.8%; thrombocytopaenia 8.3%; and elevated prothrombin time 10.4%.

Conclusions

Our results show that our RT-PCR negative patients showed signs/symptoms and abnormal laboratory values typical of COVID-19, indicating surgeons should be aware of these abnormalities in patients and the need to rule out COVID-19 before proceeding with surgery.

## Introduction

An epidemic of coronavirus disease 2019 (COVID-19) caused by infection with the severe acute respiratory syndrome coronavirus 2 (SARS-CoV-2), began in December 2019 in Wuhan, China. By the end of February 2020, hundreds of imported and resulting secondary cases were reported in several countries, and currently the pandemic is spreading rapidly across the globe, with more than 9 million infections and more than 470,000 deaths worldwide, and on March 11, 2020, the World Health Organization (WHO) declared the outbreak of this novel virus a Pandemic [1].

In Japan, the first patient with COVID-19, with a history of travel to Wuhan, was identified in our city in Kanagawa prefecture on January 15, 2020. Up to February 10, 16 COVID-19 cases were detected in all of Japan, in addition to the 696 patients with COVID-19 from the Diamond Princess cruise ship, which was anchored in the Port of Yokohama in Kanagawa from February 3 to March 1. According to a report on the Diamond Princess, among the 3,063 tested travelers, 328 asymptomatic individuals were positive for SARS-CoV-2 by reverse-transcriptase polymerase-chain-reaction (RT-PCR) by February, 20, 2020; and the proportion of the confirmed cases that were asymptomatic increased from 16.1% to 50.5% within a week from February 13 [2]. It is notable that SARS-CoV-2 infections appear to have been transmitted during its incubation in the index patient, in whom the illness was brief and nonspecific [3]. The fact that asymptomatic persons are potential sources of the infection may warrant a reassessment of the transmission dynamics of the current outbreak [4]. The Japanese government declared a state of emergency for Japanese metropolitan areas, including our prefecture, regarding COVID-19 on April 7. Citizens in our prefecture were requested to “stay home” and refrain from going out except in cases of necessity, until May 25, when the state of emergency for metropolitan areas was cancelled.

Our institution is a critical care medical centre with 1,185 beds, which is one of biggest university hospitals in Kanagawa inside the metropolitan area. We accept emergency patients including COVID-19 from secondary medical area covering 1,500,000 population within the area of 600 km2. Starting in April 6, our institution officially decided to postpone elective orthopaedic surgeries without urgent necessity, which was consistent with the statement on triage from the Japanese Orthopaedic Association (JOA). That statement followed the guidance of the United States Center for Medicare & Medicaid Services (CMS), which specified that surgeons should perform “essential” surgical procedures only and postpone or cancel “nonessential” surgery [5]. In addition, because of the long incubation period and asymptomatic spread of COVID-19, our institution decided to perform real-time RT-PCR for SARS-CoV-2, principally for all patients who required surgery. Based on these backgrounds, situation related with orthopaedic surgeries completely changed during this duration. In this study, we reviewed all the surgical cases performed during the COVID-19 pandemic from January to June, 2020 in our department. We compared the number and content of surgical cases performed during this period with the cases performed during the previous four years. We also examined the results of PT-PCR for SARS-CoV-2, which was principally performed for all the surgical candidates in our department beginning May 13, and investigated their laboratory test results before surgery, their clinical signs and symptoms, which were reported to be related with COVID-19.

## Materials and methods

Number of outpatients and surgical cases

**Figure 1:**
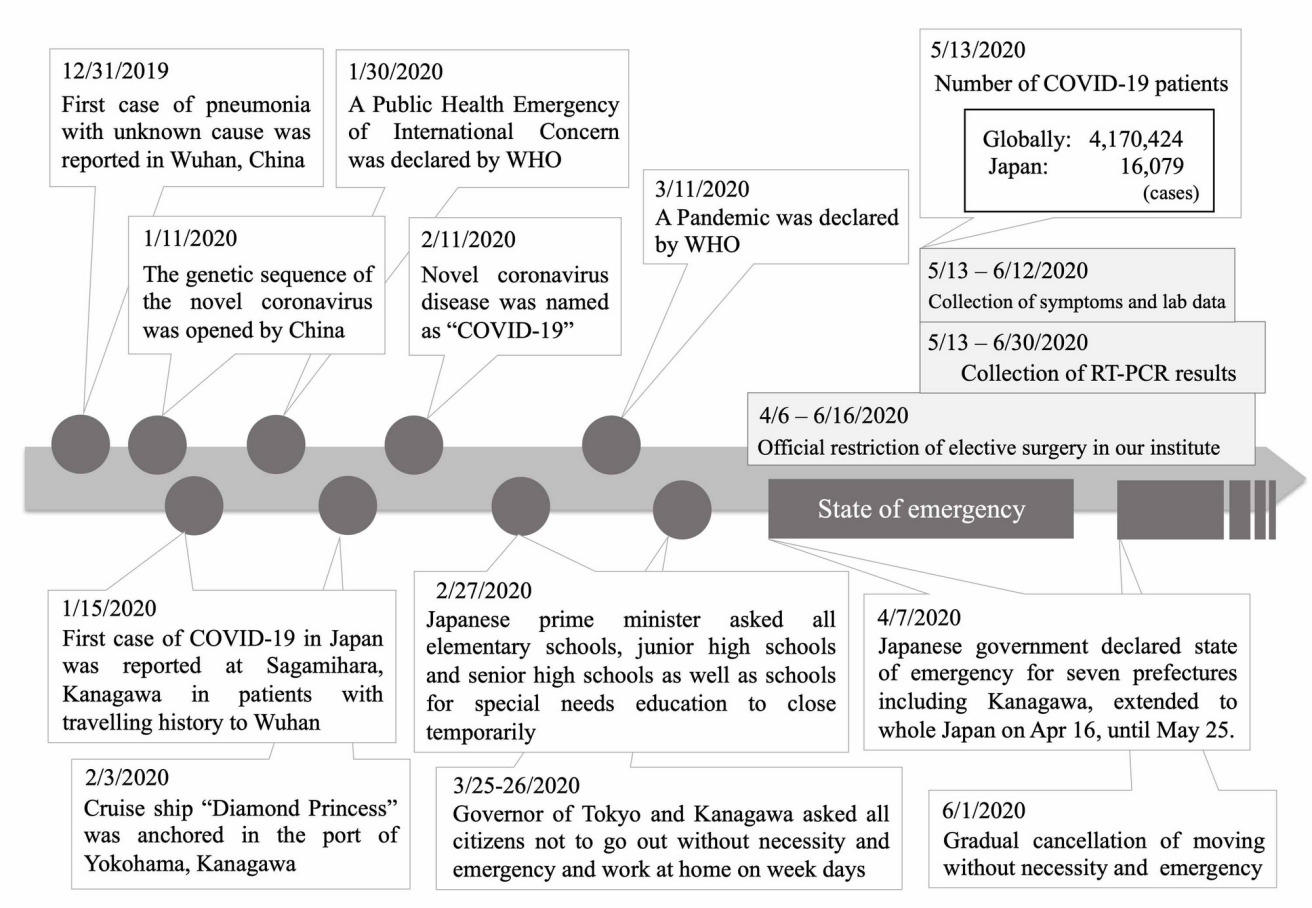
Time table of the events related to COVID-19 worldwide and in Japan RT-PCR: reverse-transcriptase polymerase chain reaction, WHO: World Health Organization

This study was approved by our institutional review board. We reviewed the monthly number of new outpatients introduced as surgical candidates and surgical cases from January 2016 to June 2020, and compared the pre-pandemic numbers with the intra-pandemic numbers of surgical cases both in elective and emergency surgery. We also investigated the relationship between the number of surgical cases and time table of the events related to COVID-19 worldwide and in Japan (Figure 1). Additionally, from January 1 to June 30, 2020, all surgical cases were reviewed, and the number of cases with frequently performed surgical methods occurring every month, and we compared the trend of change in surgical volume for each method.

Clinical signs/symptoms and results in patients with RT-PCR results for SARS-CoV-2

We undertook a retrospective review of clinical signs and symptoms in 48 consecutive patients who underwent surgery performed by orthopaedic surgeons for one month from May 13 to June 12, 2020 in our department. May 13, 2020 was the date for when RT-PCR was first used to test specimens for SARS-CoV-2, which were taken two days before surgery from every patient needing general anesthesia for their procedure. Upon admission, each patient was questioned about the presence of the following signs/symptoms typical for COVID-19 the week before surgery: fever >37.5℃, dry cough, fatigue, sore throat, dyspnoea, chest pain, nasal congestion, nasal mucus, smell impairment, taste impairment, headache, dizziness, diarrhoea, abdominal pain, nausea, and vomiting [6-9]. The incidence of these signs/symptoms was investigated, regardless of the RT-PCR results. The occurrence of signs/symptoms associated with COVID-19 during their hospital stay and the necessity of additional RT-PCR testing were also investigated.

Polymerase chain reaction assay

All patients were admitted to our institution two days before surgery and signed informed consent for RT-PCR testing. A nasopharyngeal swab was used to obtain a sample for testing by the 2019-nCoV real-time RT-PCR assay. Samples were tested for SARS-CoV-2 with the nucleic acid detection kits which were recommended by National Institute of Infectious Diseases in Tokyo. All samples were processed at the gene testing room in Kitasato University Hospital. The real-time RT-PCR was performed by a one-step method using a SARS-CoV-2 kit according to the protocol “2019-nCoV Ver. 2.8 and 2.9” both published in March 2020 by the National Institute of Infectious Diseases [10]. Primer and probe were designed to detect N and N2 genes, produced by Nihon Gene Research Laboratories Inc. (Sendai, Japan).

Collection of laboratory data

Blood tests were performed before admission, and the following laboratory data were reviewed: white blood cell (WBC) count; percentages of neutrophils, lymphocytes, and monocytes; platelet count; activated partial thromboplastin time (APTT); prothrombin time (PT); levels of D-dimer; albumin, total protein, alanine aminotransferase (ALT); aspartate aminotransferase (AST); blood urea nitrogen (BUN); and C-reactive protein (CRP); hemoglobin (Hb) A1c; and estimated glomerular filtration rate (eGFR). These laboratory data obtained from patients whose surgery was performed from May 13 to June 12 was analyzed.

Statistical analysis

Statistical Package for Social Sciences (SPSS) Statistics version 25 (IBM Corp., Armonk, NY, USA) was used for statistical analysis. Demographics were presented by mean and standard deviation (SD) or median and interquartile range (IQR), as appropriate. Categorical variables are presented as numbers and percentages. The number of cases were compared by the unpaired t-test, and a p-value <0.05 was considered statistically significant.

## Results

Number of new outpatients and surgeries in the department of orthopaedic surgery

**Figure 2:**
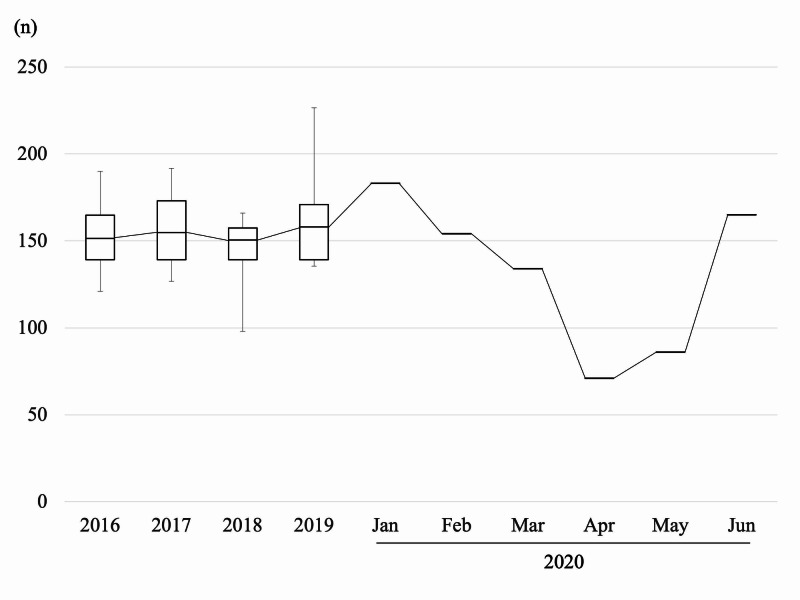
The number of outpatients per month from 2016 to 2020 Box = interquartile range (IQR), horizontal line = median, whiskers = variability outside the upper and lower quartiles.

**Figure 3:**
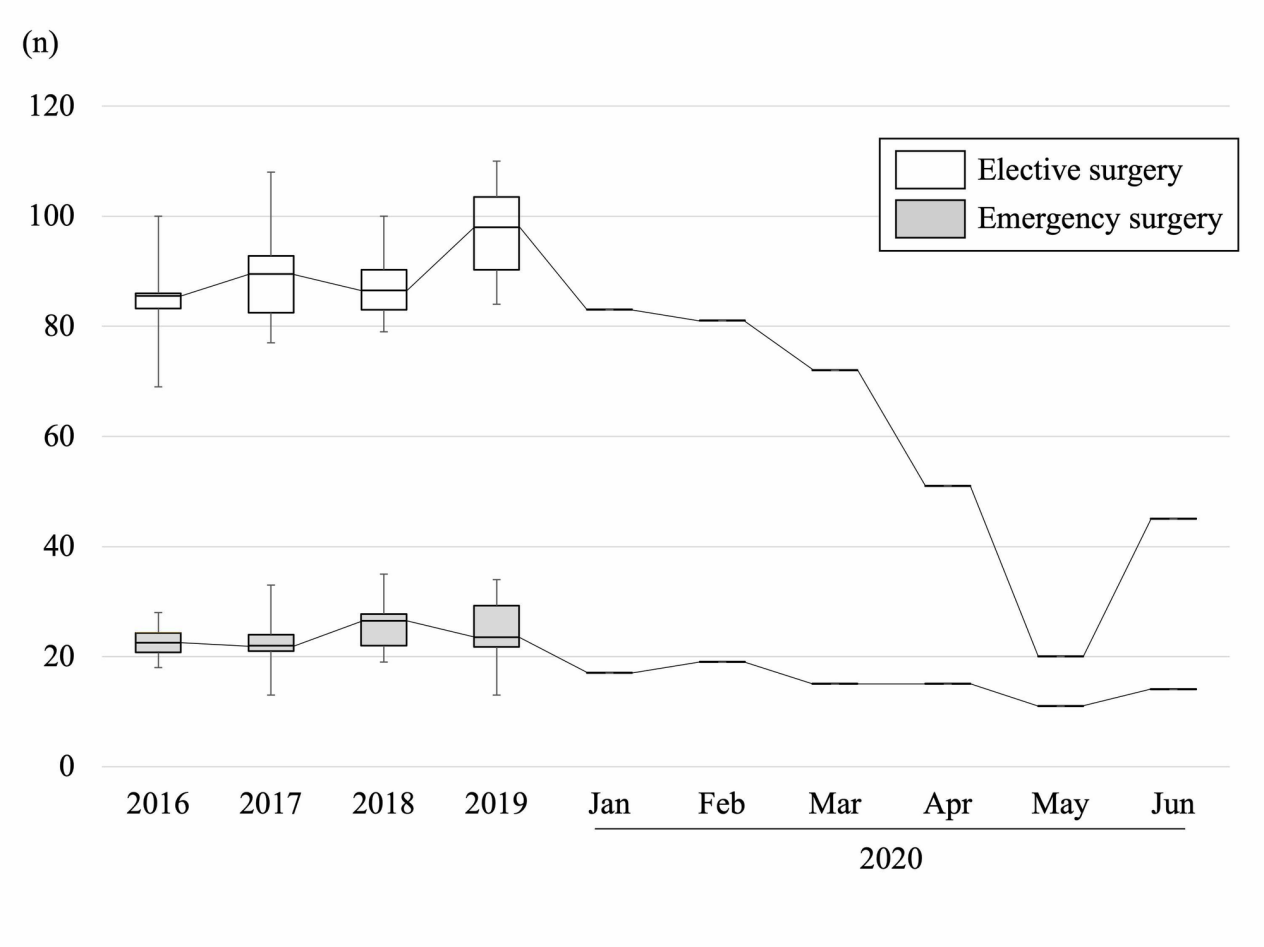
The number of surgical cases per month from 2016 to 2020 Box = interquartile range (IQR), horizontal line = median, whiskers = variability outside the upper and lower quartiles. White box: elective surgery, gray box: emergency surgery.

Figure 2 depicts the tendency in number of new outpatients to come to our clinic as surgical candidates per month. The number started decreasing in January and gradually decreased until April, and increased again in May. Figure 3 depicts the tendency in number of orthopaedic surgeries performed under general anesthesia per month. 

**Figure 4:**
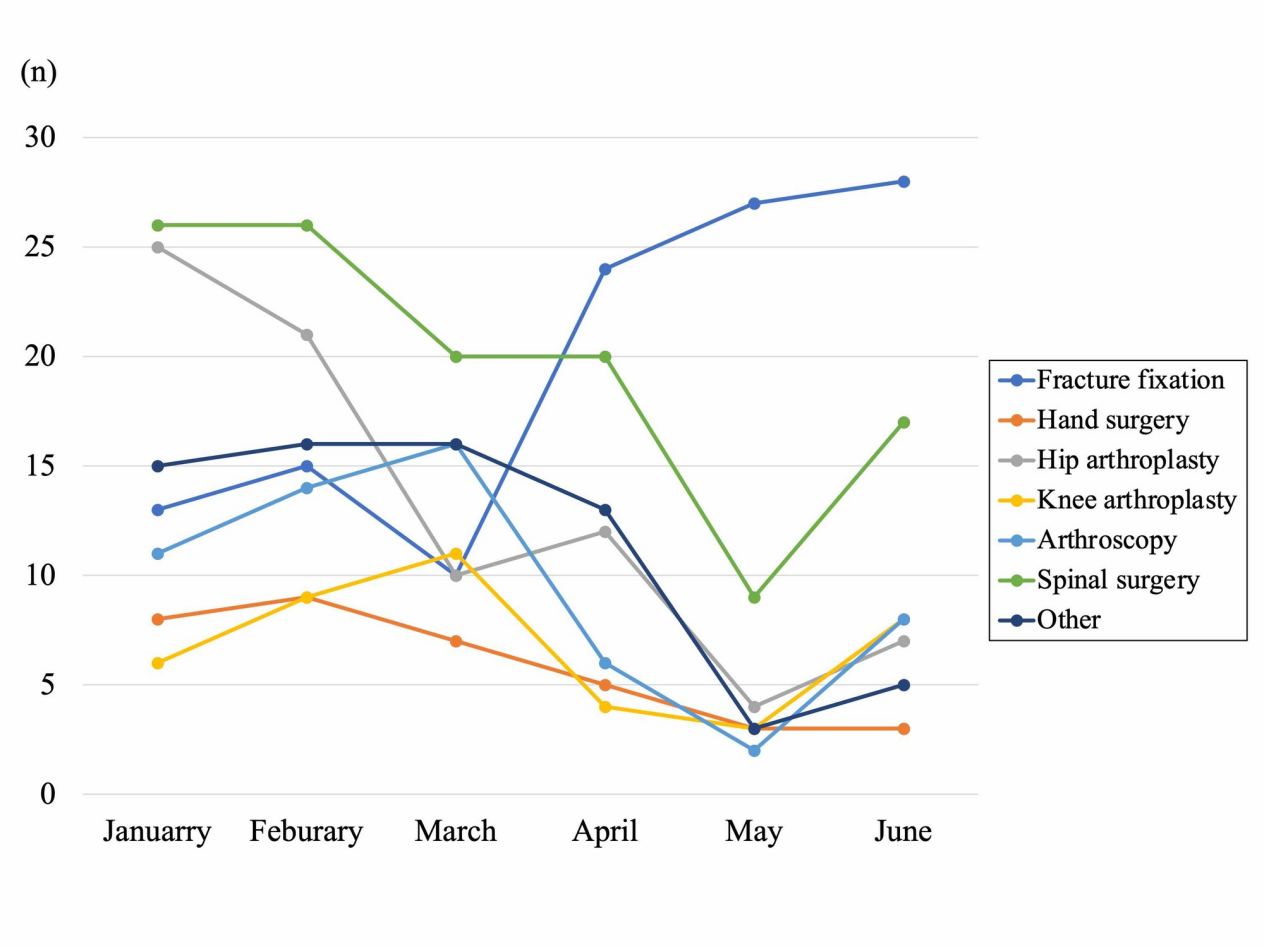
The number of representative surgical methods in 2020

Among environmental change in society as described in Figure 1, the number of elective surgeries gradually decreased from January, and decreased drastically in May. On the other hand, emergency surgery decreased gently from January to February, and did not change drastically until the end of June. The mean number of total monthly surgical cases significantly decreased from January to April 2020 than 2019 (73.8±27.1 vs 121.9±7.7, p=0.01). Compared with 2019, the reduction rate of elective surgery in May was 79.4%, which was obvious compared with the rate of emergency surgery as was 55.7%. The proportion of emergency surgery in all surgery under general anesthesia was significantly higher in May 2020 compared with the proportion in 2019 (35.5% vs 20.4%, p=0.04). Figure 4 shows the overall detail of the decreased numbers of representative surgical methods. Hip arthroplasty and spine surgery showed the most drastic change. Hip arthroplasty decreased by 50% in March, and continue decreasing by more than 80% in May. Spine surgery decreased by more than 20% in March, and showed a sudden decrease of 65% on May. Arthroscopy and knee arthroplasty decreased in April by 50% compared with pre-pandemic. Hand surgery decreased gradually, and fracture fixation did not change obviously throughout these six months. In January, the first COVID-19 patient in Japan was reported in our city, and citizens in our city started to hesitate to visit local medical institutes, resulting in gradual decrease of reference to our hospital.

Results of RT-PCR assays

A total of 95 patients receiving general anaesthesia underwent orthopaedic surgery after the initiation of RT-PCR testing until the end of June. During this period, one patient undergoing emergency surgery was not tested, based on the decision of the anaesthesiologist; because of the need for urgent procedures performed by several surgeons, without the RT-PCR testing at the later timing. A total of 94 patients were then tested by the RT-PCR assay, and none of the patients were positive for SARS-CoV-2.

Signs/symptoms before surgery

Table 1 lists the signs and symptoms of patients during the week before surgery in 48 patients, who underwent surgery from May 13 to June 12. The mean age was 61.4 years old and 19 patients were men and 29 were women. Although all patients were negative by RT-PCR for SARS-CoV-2, some signs/symptoms typical of COVID-19 were confirmed within 66.7% (n=32) of non-COVID-19 patients. The most frequent signs/symptoms were production of nasal mucus (25.5%), followed by dry cough (19.1%); and fatigue, headache, and dizziness (17.0% each). Smell impairment was reported by one patient, who was a 74-year-old man for whom shoulder arthroscopy was performed for a torn rotator cuff. He had a 10+-year history of allergic rhinitis, but an otorhinolaryngologist did not think his smell impairment was related to allergic rhinitis, and the cause of impairment remained unclear. None of the patients reported taste impairment or vomiting the week before surgery. During their hospital stay, none of the patients showed new-onset signs/symptoms of COVID-19, and none of the patients underwent PT-PCR testing.

**Table 1:** Patients' signs and symptoms within a week before admission

number	48
Sex	M:19, F:29
Age	61.4±18.3
Symptoms	% (n)
Fever	4.2 (2)
Dry cough	18.8 (9)
Fatigue	16.7 (8)
Sore throat	8.3 (4)
Dyspnea	2.1 (1)
Chest pain	4.2 (2)
Nasal congestion	14.6 (7)
Nasal mucus	25.5 (12)
Smell impairment	2.1 (1)
Taste impairment	0.0 (0)
Headache	16.7 (8)
Dizziness	16.7 (8)
Diarrhea	12.5 (6)
Abdominal pain	2.1 (1)
Nausea	6.3 (3)
Vomiting	0.0 (0)

Laboratory test results before surgery

The results of laboratory tests performed just before surgery are shown in Table 2. The mean WBC was 6.93×10^9^/L. A total of six patients had leucocytosis (>9.0×10^9^/L), 12 had neutrophilic granulocytosis (>70.0%), three had lymphocytosis (>61.4%), and 14 had monocytosis. One patient showed leukopenia with a WBC of 3.8×10^9^/L, and no patient showed lymphopenia. The platelet count was higher than the normal range in three patients (52.8, 41.4, and 34.9×10^9^/L). Blood coagulation assays showed prolonged APTT in three patients, and one patient each had a spinal metastasis from lung cancer, multiple fractures due to a fall, and secondary to warfarin before undergoing hip arthroplasty. The PT was prolonged in five patients, including two patients with spinal metastasis. Abnormal D-dimer levels were seen in 22 patients including four with 20 mg/L, two with 30 mg/L, and one with 40 mg/L. Fifteen (31.3%) of 48 patients had low albumin levels, and seven (14.6%) had low total protein levels because of their preoperative poor condition. All patients did not show severe liver or renal dysfunction. Two patients with lung cancer or multiple fractures due to a traffic accident showed a CRP level higher than 10 μmol/L. The incidence of abnormal values, which are commonly noted in COVID-19 patients were eosinopenia: 37.5%, lymphopenia: 18.8%, elevated AST: 14.6%, elevated C-reactive protein: 22.9%, elevated PT: 6.3%, elevated lactic acid dehydrogenase (LDH): 12.5%, elevated D-dimer: 43.8%, thrombocytopenia: 8.3%, and elevated ALT: 20.8%. Based on RT-PCR results, these abnormal data were not associated with findings with COVID-19.

**Table 2:** The patients' laboratory data just before surgery * a patient under hemodialysis APTT: activated partial thromboplastin time; PT: prothrombin time; ALT: alanine aminotransferase;  AST: aspartate aminotransferase; HbA1c: hemoglobin A1c; eGFR: estimated glomerular filtration rate, LDH: lactic acid dehydrogenase

Laboratory test on admission (n=48)	mean±SD (min - max)	proportion of abnormal value
		(n(%) of lower, higher level)
White blood-cell count (×10^9^/L)	6.93±3.84 (3.8 - 29.3)	1(2.1%), 6(12.5%)
Neutrophil percentage (%)	62.8±14.6 (2.9 - 85.8)	12(25.0%), 2(4.2%)
Eosinocyte percentage (%)	2.8±2.0 (0.5 - 10.7)	18(37.5%), 5(10.4%)
Lymphocyte percentage (%)	26.7±10.8 (4.8 - 61.4)	9(18.8%), 3(6.3%)
Monocyte percentage (%)	5.8±1.7 (3.0 - 12.6)	0(0%), 14(29.2%)
Platelet count (×10^9^ /L)	24.0±7.0 (11.2 - 52.8)	4(8.3%), 3(6.3%)
APTT (s)	30.4±10.4 (21.9 - 96.6)	2(4.2%), 3(6.3%)
PT (%)	102.8±19.5 (29 - 130)	0(0%), 5(10.4%)
D-dimer (mg/L)	4.40±9.07 (0.50 - 43.67)	0(0%), 22(45.8%)
Albumin (g/L)	4.09±0.60 (2.6 - 5.1)	15(31.3%), 0(0%)
Total protein (g/L)	6.98±0.67 (4.9 - 8.1)	7(14.6%), 0(0%)
ALT (U/L)	22.1±9.1 (10 - 53)	2(4.2%), 9(18.8%)
AST (U/L)	23.2±18.0 (3 - 87)	2(4.2%), 7(14.6%)
LDH (U/L)	203.3±63.5 (133 - 357)	0(0%), 5(10.4%)
Blood urea nitrogen (mmol/L)	17.3±5.8 (7.8 - 35.9)	1(2.1%), 9(18.8%)
eGFR (μmol/L)	69.9±23.8 (3* - 144)	0(0%), 2/47(28.3%)
C-reactive protein (mg/dL)	1.62±3.87 (0.03 - 20.01)	0(0%), 22(45.8%)
HbA1c (%)	5.80±0.57 (4.9 - 7.4)	0(0%), 6(12.5%)

## Discussion

**Figure 5:**
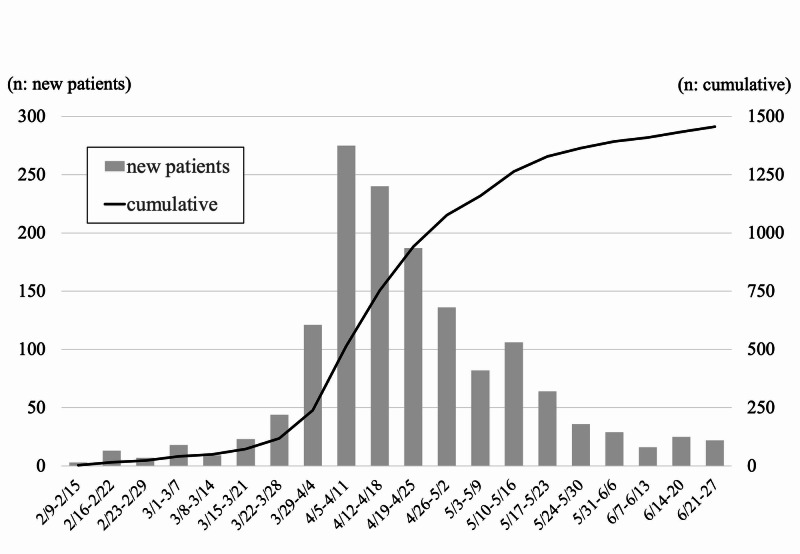
The number of patients with COVID-19 in Kanagawa prefecture Bar graph: new patients, line graph: cumulative number of patients.

The COVID-19 pandemic has changed all aspect of “normal” orthopaedic surgery in one of biggest university hospitals in Kanagawa prefecture, the capital area of Japan. During this period of the state of emergency, the Kanagawa prefecture reported that the weekly number of new patients, diagnosed with COVID-19 (excluding those associated with the Diamond Princess cruise ship) increased to a maximum of 275 new patients from April 4 to 7, and thereafter decreased gradually after the state of emergency declaration (Figure 5). In our department, we only performed less than 30% of surgery under general anesthesia in May, 2020 compared with the previous years. During this pandemic period, hip arthroplasty and spinal surgery decreased the most by 85% and 65% at maximum, followed by arthroscopy and hand surgery. The main reason of the decrease of elective surgeries from January was decrease of introduced surgical candidates from January to March, when patients started to hesitate to go to local clinics. Additionally, the petition of the prefecture not to go out without necessity and emergency, declaration of state of emergency, and the policy of our hospital to postpone elective orthopaedic surgeries without urgent necessity facilitated the intensive decrease from April to May (Figure 1). Because we accepted cases needing immediate surgery (e.g., multiple trauma, open fracture, rapidly progressing myelopathy, spinal metastasis, and pyogenic spondylitis), the cases of fracture fixation did not change throughout this period, and the emergency surgery decreased gently compared with elective surgery. On May 25, Japanese government cancelled the declaration of state of emergency, and on June 17, our institution cancelled restriction of surgical procedures, and the number of procedures recovered to about 50% of the total number of cases compared with previous years.

During this pandemic period, Japanese citizens were required to stay home and avoid going out for unnecessary or nonurgent purposes; and to avoid the “three Cs”: closed spaces with poor ventilation, crowded places with many people nearby, and close-contact settings such as close-range conversations. Additionally, our city was the first place where a COVID-19 patient was diagnosed in Japan. As a result of patient factors, the number of patients coming to the outpatient clinic as surgical candidates decreased gradually from January, and decreased drastically after the mandate by the governor not to go out without necessity and emergency at the end of March. Additionally, the JOA published guidance for triage for the postponement of nonessential orthopaedic surgery. The government mandates and guidance on surgery led our department to suppress the number of surgeries performed by our department from the middle of March to the end of June, apart from patients' factor. In the United States, over 30,000 hip and knee arthroplasty were estimated canceled each week during restrictions established because of the COVID-19 pandemic [11]. A large cohort study of 7.5 million residents in Hong Kong reported that orthopaedic surgeries were reduced by 44.2%, and elective joint replacements and ligament reconstructions were decreased by 74% and 84%, respectively [12]. The number of surgeries in our institute reduced by 85% in hip arthroplasty and by 70% in total. Content of surgical methods should vary depending on the characteristics of each institute, and the reduction rate was consistent to the previous report.

RT-PCR was positive for SARS-CoV-2 in 0% of our 94 consecutive cases from May 13 until June 30. Keio University Hospital in Tokyo started to perform the 2019-nCoV real-time RT-PCR assay at their outpatient clinic on April 6 for all patients who needed admission. Their press release reported the incidence of RT-PCR positivity in asymptomatic patients every week from April 6 thereafter. The highest incidence of RT-PCR positivity was 7.5% (five of 67 patients) during the week of April 13 to 19 [13]. In New York City, RT-PCR assays of nasopharyngeal swab specimens from 99 patients undergoing routine testing for SARS-CoV-2 prior to their planned orthopaedic surgical procedure showed a positivity rate of 12.1%. A total of 58.3% of the positive specimens were from asymptomatic patients [14]. These results suggest that there are unknown numbers of asymptomatic patients who could be potential sources of an outbreak of COVID-19 in any hospital elsewhere. The rates of asymptomatic individuals might vary between regions or types of hospitals and our results could not be consistent in another region even in Japan.

In Japan, some experts thought that the indications for RT-PCR testing were too narrow, and the daily number of people tested was too low, resulting in an inaccurate number of individuals with COVID-19. Indeed, the rate of RT-PCR testing in Japan has been reported to be 2.2 per 1,000 people, which is the 36th lowest among 37 countries participating in the Organization for Economic Co-operation and Development (OECD) [15]. However, the data from the Tokyo Metropolitan government showed that the actual positive rate also decreased from 31.7% on April 11 to 1.8% on May 13, and did not increase to higher than 2.0% up until June 15 [16]. These epidemiologic data from Kanagawa and Tokyo suggest that the first wave of COVID-19 might have peaked in the metropolitan area in early June.

In COVID-19 patients with pneumonia and abnormal findings on chest computed tomography, the reported signs/symptoms at onset included fever (98%), cough (76%), myalgia or fatigue (44%), sputum production (28%), headache (8%), haemoptysis (5%), and diarrhoea (3%); and after a median time of eight days from the onset of illness, dyspnoea developed in 55% of 41 patients [17]. The other report showed that impairment of smell or taste was often the first apparent symptom, with a prevalence of 64.4% in 202 mildly symptomatic patients, but that it rarely was the only symptom of SARS-CoV-2 infections [18]. Schneider et al. evaluated 66 orthopaedic healthcare workers exposed to one patient who became positive for SARS-CoV-2 infection one week after admission, and reported that the RT-PCR assays were negative for all 66 healthcare workers, although 14 (21%) manifested clinical signs/symptoms suggestive of COVID-19, including cough (6.1%), sore throat (4.5%), nasal congestion (4.5%), dyspnoea (3.0%), fever (1.5%), headache, and myalgias (1.5%) [19]. In our study, although PT-PCR testing was negative for all consecutive 48 patients, several signs/symptoms were noted, including nasal mucus (25.5%), cough (19.1%), fatigue (17.0%), headache (17.0%), dizziness (17.0%), nasal congestion (14.9%), and diarrhoea (12.8%). A few patients also reported sore throat, nausea, fever, chest pain, dyspnoea, abdominal pain, and smell impairment. These symptoms are not specific to COVID-19 and have been confirmed to occur in patients undergoing orthopaedic surgery at various frequencies.

There are several papers that revealed the characteristics of laboratory data of COVID-19 patients. Among 41 patients with COVID-19 related pneumonia, 25% had leukopenia (WBC less than 4×10^9^/L) and 63% of patients had lymphopenia (lymphocyte count less than 1.0 × 10^9^/L) [17]. A literature review reported that common COVID-19 laboratory findings include eosinopenia (frequency noted in COVID-19 patients: 78.8%), lymphopenia (68.7%), elevated AST (63.4%) levels, elevated CRP (60.7%) levels, elevated PT (58.0%), elevated lactate dehydrogenase (LDH) (47.2%) levels, elevated D-dimer (46.4%) levels, thrombocytopenia (36.2%), elevated ALT (21.3%) levels, and elevated high-sensitivity troponin (12.5%) levels [20]. In this study, only one patient (70-year-old man) with multiple myeloma showed leukopenia (WBC 3.8 × 10^9^/L), and the mean D-dimer level of patients was relatively higher (4.4±9.1 mg/L). In our patients, only the incidence of elevated D-dimer levels (43.8%) was similar which might be caused by various patients' backgrounds including severe general condition, but the other parameters were lower than those of the COVID-19 patients noted in the literature review. Our results suggest that abnormal values in laboratory data themselves were not specific for COVID-19, but surgeons should always be aware of the possibility of abnormal laboratory data that are uncommon in the usual clinical setting of orthopaedic surgery to rule out COVID-19 patients.

Now, at the end of June, the time of this writing, the weekly number of new COVID-19 patients in Kanagawa prefecture seems to be under control (Figure 5), but citizens were allowed to move across prefectures and increasing tendency are noted, with several clusters in Tokyo. Also, the cases of orthopaedic surgery were increasing now. We will continue RT-PCR testing for SARS-CoV-2, in preparation for the second wave of infections that should arrive in the near future, trying to resume adequate orthopaedic surgeries. Further evidence of experiences related with COVID-19 is essential to establish the appropriate medical system to produce the orthopaedic surgery properly in this era of coexistence with the COVID-19.

## Conclusions

Our results show that, in 2020, the number of elective surgeries gradually decreased from January until May, but emergency surgery decreased gently from January to February, and did not change drastically until the end of June in our hospital. Although none of the patients were positive for SARS-CoV-2 during the first wave of COVID-19 in our department, 66.7% of the patients showed signs/symptoms typical of COVID-19, indicating surgeons should be aware of these abnormalities in patients and the need to rule out COVID-19 before proceeding with surgery.
